# Human Teaching and Cumulative Cultural Evolution

**DOI:** 10.1007/s13164-017-0346-3

**Published:** 2017-06-14

**Authors:** Christine A. Caldwell, Elizabeth Renner, Mark Atkinson

**Affiliations:** University of Stirling, Stirling, FK9 4LA UK

## Abstract

Although evidence of teaching behaviour has been identified in some nonhuman species, human teaching appears to be unique in terms of both the breadth of contexts within which it is observed, and in its responsiveness to needs of the learner. Similarly, cultural evolution is observable in other species, but human cultural evolution appears strikingly distinct. This has led to speculation that the evolutionary origins of these capacities may be causally linked. Here we provide an overview of contrasting perspectives on the relationship between teaching and cultural evolution in humans, and briefly review previous research which suggests that cumulative culture (here meaning cultural evolution featuring a trend towards improving functionality) can occur without teaching. We then report the results of a novel experimental study in which we investigated how the benefits of teaching may depend on the complexity of the skill to be acquired. Participants were asked to tie knots of varying complexity. In our Teaching condition, opportunities to interact with an experienced partner aided transmission of the most complex knots, but not simpler equivalents, relative to exposure to completed products alone (End State Only condition), and also relative to information about the process of completion (Intermediate States condition). We conclude by considering the plausibility of various accounts of the evolutionary relationship between teaching and cultural evolution in humans.

## Introduction

The role of culture in human behaviour has no parallel in any other species, and it is responsible for many of the characteristics which distinguish us from even our closest evolutionary relatives (Hill et al. 2009; Tomasello 1999). By definition, culture involves the social transmission of information from one individual to another. There is therefore widespread interest from behavioural scientists in the specific role that teaching may play in this process, and the extent to which it may account for the apparently unique features of human culture. Here, we provide an overview of the contrasting accounts of the relationship between teaching and human-specific culture, and consider evidence which provides insights into the role of teaching in cumulative culture. We first introduce the concepts under discussion.

### Cumulative Cultural Evolution

Cultural evolution refers to situations in which the behaviours or behavioural artefacts of a population exhibit changes over time as a consequence of social transmission. This can be due to either contributions from individual learning processes, errors in transmission, or both. Thus defined, cultural evolution has been convincingly demonstrated in nonhumans (e.g. birdsong: Slater 1986; vocal dialects in cetaceans: Ford 1991) as well as humans.

Cumulative cultural evolution is often defined as a specific case of cultural evolution, where such changes exhibit a directional trend, typically representing improvements upon, or elaborations of, earlier alternatives. Cultural evolution of this kind is universally recognised to be widespread in humans, since the existence of learned behaviours that could not have been invented by a single individual provide unambiguous evidence. Although putative examples of possible cumulative culture have been identified in wild populations of nonhumans (e.g. chimpanzees: Sanz et al. 2009; New Caledonian crows: Hunt and Gray 2003), such examples are noteworthy for their rarity. Furthermore it should be emphasized that the interpretation of these cases remains highly controversial. This is largely due to the absence of relevant historical data which would have the potential to confirm the past prevalence of less effective behavioural variants which could have acted as antecedents to the present dominant form. Additionally, in some instances, the role of social transmission in the acquisition of the behaviour may not yet be firmly established (e.g. Kenward et al. 2005). A recent experimental study of cultural evolution in baboons (Claidière et al. 2014) is similarly controversial as a possible example of cumulative culture as we have defined it here, and could be argued to have no recognisable parallel in any naturally occurring behaviour. Claidière et al. (2014) trained baboons to reproduce patterns on touchscreen monitors, and showed that iterative transmission of the patterns produced by the baboons (as target stimuli for successive experimental subjects) resulted in striking regularities common to many of the response patterns that emerged over the transmission process. This was presumably due to fact that the baboons’ errors in reproduction tended to transform patterns in non-random ways.

By contrast, human populations across the globe are universally reliant on an abundance of traits that are unambiguously an outcome of cumulative cultural evolution, with many of those representing essential survival skills (Henrich and McElreath 2003). Much of human collective expertise and technology is known to have arisen as a consequence of many generations of accumulated inventions, with novel developments typically dependent on earlier discoveries in an ongoing iterative process. Human cumulative culture has been described as operating like a “ratchet” in this respect (Tomasello 1990): an analogy for the apparently inexorable escalation of cultural traits. It is perhaps unsurprising therefore that considerable effort has been devoted to offering possible explanations for this peculiarly human characteristic, and we review these in Section 1.3.

### Teaching in Humans

Kline (2015) has defined teaching as, “behaviour that evolved to facilitate learning in others”. This definition is specific and concise, and hence we believe it is a very useful one. Thornton and Raihani’s (2008) definition of teaching as: “a form of cooperative behaviour which functions to promote learning in others” is similarly useful. However, since we are currently interested in seeking clarity regarding what seems to be distinctive about human teaching compared with widely accepted examples of teaching in animal behaviour, we feel that it is necessary to note that we believe that neither of these fully captures all cases of human teaching (including many that would readily be labelled as such by any typical layperson). In placing the focus on evolution (Kline) and function (Thornton & Raihani), these two definitions readily accommodate all cases in which the behaviour has been subject to any kind of outcome-driven selection (potentially including either genetic or cultural evolution). However, in many examples of human teaching behaviour, there has been no such feedback, which also means that the “teaching” may not necessarily function to facilitate learning. Rather, the teacher engages in the behaviour as a consequence of their own belief (which may or may not be substantiated) about the likely effect on the learner.

Thus, teaching can occur as a consequence of either a history of successful function, and shaping by selective processes, or an intention to transmit knowledge. And although these are far from being mutually exclusive, we believe that highlighting the distinction between intentional design and design by selective processes provides a helpful clarification of important differences between examples of teaching behaviour in nonhumans and the vast majority of what would be widely regarded as teaching in humans. We elaborate on this in the remainder of this section.

In contrast to cumulative culture, there are multiple widely accepted examples of teaching behaviour in nonhumans (e.g. tandem running in ants, Franks and Richardson 2006; provisioning of prey in meerkats, Thornton and McAuliffe 2006). The demonstrable benefits to learners and costs to teachers strongly suggest that the behaviours have been selected as a consequence of their effect on others’ learning (in line with Caro and Hauser’s 1992, operational criteria for teaching, and also Kline’s 2015, definition). Consequently, discussion of human uniqueness in relation to teaching has focused not on the question of the presence or absence in nonhumans, but on attempting to capture the nature of the distinction between human-specific teaching and teaching in other species.

Kline (2015) has proposed a taxonomy for teaching behaviour which includes a category of *direct active* teaching. Within this framework, direct active teaching is characterised by a selective emphasis on information relevant to the learner, and the learner’s interpretation of this information. Kline (2015) noted that to date no clear examples of this kind of teaching have been identified in nonhumans. Csibra and Gergely (2009) have contended that humans are the only species to transmit generalizable knowledge through communication. In considering the evidence from nonhuman species, Csibra (2007) asserted that tandem running in ants transmitted only episodic – not generalizable – information, and that prey provisioning in meerkats was an example of scaffolding, rather than communication. Thornton and Raihani (2008) have highlighted the flexibility of human teaching as being a key feature not shared by other species. They attributed this flexibility to humans’ ability to attribute knowledge, which allows teachers to: “generalize across contexts, to recognize ignorance in their pupils and to alter their techniques according to individual pupils’ current knowledge, rather than simply responding to behavioural cues”.

There is broad consensus therefore that human teaching is particularly responsive to the needs of the learner, whereas examples of teaching behaviour in nonhumans appear much more rigid and constrained. We believe that to date there has been no evidence of nonhuman behaviour that is designed to teach as a consequence of intention rather than selection on functional outcomes. This places inherent constraints on nonhuman teaching since it is likely to be restricted to contexts which have an extended evolutionary history. Nonhuman teaching may therefore be observed for common species-typical behaviours, but this is unlikely to extend to the transmission of behavioural innovations. This kind of teaching behaviour (i.e. which fulfils a teaching function, but which the teacher engages in without awareness of this function) likely occurs in humans as well (infant-directed speech may be just such an example, e.g. Eaves et al. 2016). However, the distinctively human predisposition for intentional teaching is likely to be particularly useful for the transmission of cumulative culture, as this involves an ongoing process of innovative modification, which gives little opportunity for context-specific teaching behaviours to evolve via selection.

### Views on the Relationship between Teaching and Cumulative Culture

There is an obvious appeal to accounts of human uniqueness which propose that the existence of any noteworthy human peculiarity can be explained as the consequence of another. However, doing so in the case of human culture and human teaching presents a chicken-and-egg problem. This is highlighted by consideration of some of the views expressed in the existing literature. Those interested in cumulative culture often attribute its existence (at least in part) to a human proclivity for teaching. Conversely, those interested in the universality and distinctiveness of human teaching generally view this as a consequence of the need to transmit complex cultural behaviours. We consider these perspectives in turn, as well as some additional alternatives.

We wish to emphasise that in the following section we aim to characterise some possible evolutionary scenarios, and use examples from certain authors’ writing to illustrate these. We do not intend to imply that any author or group of authors are unequivocally committed to one of these scenarios to the exclusion of all others. To our knowledge, most researchers working in this field (like us) regard these issues as unresolved, and indeed they have in all cases made efforts to emphasise the speculative nature of the accounts they have proposed.

#### Cumulative Culture as a Consequence of Teaching

Following their study of puzzlebox solution learning in groups of children, chimpanzees, and capuchin monkeys (Dean et al. 2012), Dean et al. (2014) proposed that human cumulative culture arose as a consequence of a “package” of socio-cognitive capabilities (including teaching, imitation, verbal instruction and prosocial behaviour) facilitating high-fidelity information transmission. Dean et al. (2012) found that the more advanced (multi-step) solutions to their puzzlebox task were transmitted in groups of children, but were not transmitted within the nonhuman primate groups they studied, and that children engaged in high levels of imitation, teaching, and sharing behaviour, which were not observed in monkeys or chimpanzees. Based on these findings, Dean et al. (2012) stated that: “These findings pave the way for an exciting avenue of research into when and why this particular ‘package’ of other-regarding sociocognitive capacities evolved.” The uncertainty about the “why” gives a clear indication that in this instance these authors are suggesting that cumulative culture is more likely to be consequence, rather than cause, of this evolutionary development.

Tennie et al. (2016) have presented a similar proposal, building on accounts proposed by Tomasello and colleagues (e.g. Tomasello et al. 1993) which emphasise the importance of particular social learning mechanisms which facilitate high fidelity transmission of behaviour, preventing loss of rare or subtle modifications and improvements. Tennie et al. (2016) proposed that particular modes of social learning may have predated the existence of cumulative culture in humans, and contend that the former are essential preconditions of the latter: “It is possible that the specific adaptations for human cumulative culture (especially motivation and skill in complex forms of teaching and imitating … ) existed for hundreds of thousands of years before the appearance of the suite of conditions that are favourable for cumulative culture … [F]or early hominins (and perhaps even for modern day great apes) … – perhaps – the general ability for cumulative culture was and is present, but that it was/is rarely or never expressed.” (Tennie et al. 2016, p129). Although the specified adaptations are perhaps not best described as being “for” cumulative culture in this context, otherwise this represents a clear account of a scenario under which capacities for teaching already existed, and were then exapted for the transmission of complex cultural behaviours which appeared much later.

#### Teaching as a Consequence of Cumulative Culture

Taking a different perspective, Gergely and Csibra (e.g. Gergely and Csibra 2006; Csibra and Gergely 2011) have proposed that human propensities for social learning and teaching evolved in response to the increasing “opacity” of hominin culture (i.e. behaviours for which the purpose and/or causal relevance may not be obvious to an observer). Human teaching was therefore: “designed ‘for’ the learner to guide and constrain his inferential attempts to identify … the new and relevant cultural contents to be acquired.” with learners correspondingly “equipped with specialized cognitive devices to infer and fast learn the relevant and new cultural information demonstrated ‘for’ them” (Gergely and Csibra 2006). This account is therefore very explicit about the fact that human culture must have developed to a level of complexity not present in other species first, with the evolution of human-specific teaching capacities following on from this.

Sterelny (2012) has made a similar argument, proposing that, “information-rich traditions might become quite important without any change in individual cognitive equipment”, but that such traditions would then create, “selection in favor of mutations that increase the reliability and accuracy of learning from the parental generation” (including teaching). Fogarty et al. (2011) also make a case for cumulative culture creating selection pressure favouring the evolution of teaching in humans. Their theoretical genetic models indicated that teaching would be favoured under a highly restricted range of circumstances, explaining its rare and sporadic appearance amongst nonhuman animals. However, the value of teaching was found to be considerably enhanced (and its evolution therefore favoured under a much wider range of conditions) when cumulative culture was present.

An appealing alternative interpretation is that teaching is itself culturally acquired, rather than being the direct result of biological adaptation (Heyes 2012). Heyes (2012) argues that teaching is transmitted via social interaction, and that "features that could be acquired in the course of development, and that co-vary flexibly with sociocultural experience" support such an account. Once acquired, teaching may then have the subsequent potential to aid the transmission of "facts about the world and how to deal with it", i.e. other traits of cumulative culture. According to this account, teaching is not so much a consequence of cumulative culture, but an instance of it.

#### Coevolutionary Accounts of Teaching and Cumulative Culture

Boyd et al. (2011) have proposed that mutually reinforcing effects resulted in the escalation of both complex culture and propensities for high-fidelity transmission: “The evolution of the psychological capacities that give rise to cumulative cultural evolution is one of the key events in our evolutionary history. The availability of large amounts of valuable cultural information would have favoured the evolution of bigger brains equipped to acquire, store, organize, and retrieve cultural information, a fact that may explain the rapid increase in human encephalization over the last 500,000 y and the evolution of specialized cognitive abilities that emerge early in life, such as theory of mind, selective social referencing, [and] overimitation […]” (p10924). The initial “psychological capacities” are apparently intentionally non-specific in this account, with more specialized competencies adapted for cultural transmission appearing only once these were made advantageous by the existence of a significant body of cultural expertise worth learning.

It should be noted that this particular account makes no explicit mention of teaching (in its peculiarly human form) as either precursor of, or adaptive response to, the existence of enhanced culture. Nonetheless, Boyd et al.’s (2011) argument can be readily extended to encompass teaching as one of the important forces within this coevolutionary process of mutual reinforcement. This is certainly highly plausible as an evolutionary scenario. Indeed, although the other accounts we have summarized above emphasize the influence of one factor on the other, underlying most of these is an assumption (either explicit or implicit) of some degree of mutual influence, and coevolution of the two traits.

Similar arguments have been put forward by Morgan et al. (2015) and Zwirner and Thornton (2015), both of whom have argued that relatively simple cultural contents probably generated the initial selection pressure for specialised social transmission mechanisms (including teaching), and that a positive feedback process was likely to have ensued, whereby the complexity of cultural behaviours, and the mechanisms used to transmit these, continued to escalate.

In relation to our current question of interest, coevolutionary accounts could potentially benefit considerably from additional theoretical and/or empirical evidence which might help to flesh out the details of an evolutionary timeline of likely relationships between degree or manner of cultural complexity and behavioural tendencies required to facilitate transmission.

#### Cumulative Culture and Flexible Teaching as Common Consequences of Other Causal Influence

There is of course an alternative evolutionary scenario which would also account for the coexistence of teaching and cumulative culture in humans alone, without requiring that either one of these traits triggered the other. It is entirely possible that a third causal influence enabled both human-unique forms of teaching, *and* cumulative cultural evolution as we see it in human populations. The two may or may not have reinforced one another (see previous section) but either way it is possible that neither was a strict prerequisite to the other.

There could of course be a range of plausible proposals of this kind, each implicating slightly different fundamental capabilities with the potential to independently support both teaching and cumulative culture in humans. We believe that a particularly compelling argument can be made concerning particular social metacognitive capacities. Explicit awareness of others’ mental states (or *theory of mind*, exemplified by passing the unexpected transfer false belief test, e.g. Wimmer and Perner 1983; Call and Tomasello 1999), could have brought about radical changes to the ways in which humans were able to both use information gleaned from others and facilitate the learning of others.

Regarding the facilitation of others’ learning, it is obvious how such a capacity would alter one’s behaviour; it would allow a teacher to model and predict the knowledge state of a learner, and to use this information to guide their own behaviour in a manner intended to facilitate transmission of knowledge and skills. We have already alluded to the fact that *intention* to teach may be a key distinction between human and nonhuman teaching behaviour, allowing the flexibility to facilitate learning of novel traits, as well as those that have long existed within the repertoire of the species (see Section 1.2).

In addition, it has been argued that explicit metacognitive capacities may have enabled human cumulative culture, as a consequence of the ability to devote “Type 2/System 2” cognitive processes (e.g. Evans and Stanovich 2013) to the task of evaluating others’ knowledge. Type 2, or System 2, cognitive processing has been characterised as slow, serial, dependent on working memory, and accessible to awareness, supporting rule-based, hypothetical decision making. Type 1 (or System 1) processing, in contrast, is considered to be largely dependent on autonomous associative processes which operate rapidly and without conscious access. Although Type 1 processing exhibits parallels with much of animal cognition, Type 2 appears to be particularly well-developed in humans.

This would allow the learner to accurately estimate the degree of benefit potentially available from following the example of particular individuals, based on what is known, or can be inferred, about the experience of those individuals. Heyes (2016a) has provided a detailed account of such a proposal. Heyes (2016a) account includes consideration of the possibility that “social learning strategies” identified in nonhumans may be largely the outcome of associative learning processes which, whilst producing behaviour which might appear superficially equivalent to human metacognitive strategies, would offer markedly less flexibility. Thus, representing others’ knowledge broadens the scope of both what can be learned as well as what can be taught, offering a potential explanation for both the apparent open-endedness of human cumulative culture as well as the unconstrained nature of human teaching.

It is important to highlight the distinction between accounts such as the one we wish to exemplify here, and those detailed in Section 1.3.1, which also tend to implicate theory of mind. The accounts detailed in 1.3.1 are focussed on the importance of high fidelity social transmission for sustaining cumulative culture, and theory of mind may play a supporting role inasmuch as it permits intentional teaching, and might also facilitate the copying of others’ behaviour via imitation. Cumulative culture would therefore be dependent on sociocognitive abilities to the extent that these facilitate high fidelity transmission (with teaching as one example of this). In contrast, within the current account, it would be theoretically possible for cumulative culture and teaching to exist independently of one another, or to have evolved in the absence of the other. However, they might still be expected to co-occur if they shared a common reliance on underlying cognition. Cumulative culture, on this view, would arise primarily as a consequence of the decision processes informing copying (and importantly, also selectiveness and/or restraint in copying), as opposed to copying per se.

We should also note that an account along these lines does not actually solve the chicken-and-egg problem described previously. Instead of querying the origins of cumulative culture and teaching we are left querying the origins of explicit metacognition. And indeed, Heyes and colleagues (e.g. Heyes and Frith 2014) have argued elsewhere that explicit mind-reading may well be an outcome of cultural evolution. Following this logic, with cultural evolution and explicit theory of mind posited as both cause and consequence of one another, there is also an alternative co-evolutionary scenario, which in this instance implicates only cultural evolutionary processes, rather than gene-culture interactions (as in the account described in Section 1.3.3).

### Investigating Prerequisites of Cumulative Culture Using Microsociety Design Experiments

It is difficult to resolve the question of the manner in which these capacities may have impacted on one another during human evolution, and which (if either) represented the fundamental precursor that enabled the other. This is because we simply do not have any way of accessing the relevant historical information. However, experimental methods designed to elicit cumulative cultural evolution under laboratory conditions have provided a means by which researchers can at least attempt to investigate the prerequisites of this process.

Caldwell and Millen (2008) established that it was possible to demonstrate cumulative cultural evolution under laboratory conditions, using a replacement microsociety approach. These microsocieties were essentially miniaturised populations of learners, in which generational succession was simulated through the repeated removal and replacement of individual members. In this way it was possible to capture the characteristic “ratcheting” of human culture on a small scale. Across two different experimental tasks (maximizing flight distance of a paper airplane, and maximizing height of a tower constructed from spaghetti and modelling clay), Caldwell and Millen (2008) found improvement in performance over successive attempts. This occurred in spite of the fact that each attempt was made by a different individual, and each individual had the same amount of time to complete the task. Participants in later generations performed better in terms of the goal measures, consistent with the retention of beneficial modifications and accumulation of expertise over generations, and therefore indicative of cumulative culture.

Using these methods Caldwell and Millen (2009) were able to test predictions concerning the necessary conditions for cumulative culture, through the manipulation of the information available to learners in these microsocieties. Using the paper airplane task once more, Caldwell and Millen (2009) demonstrated that adult human participants were capable of generating cumulative culture even when restricted to information about results and end products alone; neither active instruction nor action copying were necessary for this to occur.

However, in line with the proposals of Csibra and Gergely (2011) detailed previously, there is good reason to believe that teaching will be particularly useful for the transmission of relatively opaque cultural knowledge. By contrast, the task used by Caldwell and Millen (2009) was likely to represent a highly transparent skill, since the actions required to produce the end product could be determined through inspection of the end product alone.

Subsequent studies employing similar methods to Caldwell & Millen’s provide some support for the belief that different sources of information may be required for the transmission of less transparent skills. For example, Wasielewski (2014) emphasised the importance of imitation for cumulative culture involving cognitively opaque behaviour, but the argument potentially applies equally well to teaching. In Wasielewski’s (2014) study participants created structures that supported weights, some of which would have been difficult to reverse-engineer as internal structures were not always visible. There were other significant differences in approach between this study and Caldwell and Millen’s (2009) however (e.g. lack of feedback to participants about the relative efficacy of others’ structures), so conclusions should be drawn with caution. However, Zwirner and Thornton (2015) used a design very similar to Caldwell and Millen’s (2009) but involving a different task (making baskets for transporting quantities of rice). They found some evidence that teaching provided benefits over and above learning from end products alone, although both conditions showed evidence of cumulative improvement in performance. In addition, Morgan et al. (2015) investigated the transmission of stone tool making skills and found that these were more effectively preserved within chains that were allowed to use teaching and linguistic communication, compared with chains in which only imitation or end product copying was possible. Morgan et al.’s (2015) study did not actually look at cumulative culture, since the measure of interest concerned the degree of loss involved in the transmission of a target skill. Nonetheless it follows that for certain behaviours, effective transmission may only be possible with the help of teaching, and that this would therefore be necessary for cumulative culture to occur.

Consequently, it is possible that the utility of teaching may vary in accordance with ease of reproduction for skills that are matched in other respects. This would also be in line with theoretical literature on this topic, which has asserted that teaching offers benefits only when skills cannot be readily acquired through either individual learning, or learning from inadvertent social information (e.g. Thornton and Raihani 2008; Fogarty et al. 2011). We should therefore expect to find that teaching tends to be particularly effective in such contexts, although to date this has yet to be empirically demonstrated. The study reported below aimed to provide insights into this possibility.

## An Experimental Investigation of the Benefits of Teaching for Transmission of Simple and Complex Knot-Tying Skills

### Rationale

The aim of this experiment was to identify whether human-specific features of teaching offered some benefit to transmission of a novel skill, and if so, whether such an effect was more pronounced for skills that were more complex, compared with simpler equivalents. In particular, we were interested in the capacity for anticipating and responding to the needs of the learner flexibly in real time. We therefore operationalised teaching (in a Teaching condition) such that naïve learners were given opportunities for coordinated interaction with experienced individuals who were tasked with attempting to pass on their skill. We also ran two control conditions to help isolate the extent to which human-specific features of teaching were effective over and above other sources of information potentially available from incidental exposure to the behaviour or products of an experienced individual’s activity. The End State Only condition was intended to establish the effect of learning from the end products alone (“emulation” sensu Wood 1989), and the Intermediate States condition was intended to determine the effect of learning from limited process information, such as one might obtain from eavesdropping on an experienced individual engaging in the target activity.

In addition, to establish the value of instruction relative to the degree of complexity of the skill, several different behavioural products were used as target stimuli. The target solutions all represented examples of a relatively well-defined category (knots), thus permitting fairly direct and valid comparisons of relative difficulty. Two of these examples were selected specifically for the relative simplicity of their completion, and two for their difficulty. It was assumed that the process of completion for the more difficult knots would be more opaque to learners, due to the number of non-intuitive sub-goals involved in completion. Knot-tying has formed the focus of previous experimental studies of cultural evolution for reasons similar reasons to ours (i.e. due to the complex and opaque nature of the actions required). Derex et al. (2013) found that groups that could share information about process of completion, outperformed groups that could only share information about completed products, in a virtual knot-tying task. Muthukrishna et al. (2014) found that expert-taught knot-tying skills survived fewer generations of social transmission, when the transmission occurred between single individuals (one participant per generation) compared with transmission between cohorts consisting of multiple potential models (five participants per generation).

We predicted that, relative to the control conditions, the Teaching condition would be particularly beneficial for learners assigned the more difficult knots.

### Method

#### Participants

One hundred and fifty-five undergraduate students (115 female) took part in the study as part of a psychology practical class at [name of institution removed due to blind review requirement]. During the Test Trial (see Procedure), 40 participants (31 female) were assigned the role of teacher, and 115 (84 female) took part as learners. The study was approved by the University’s Psychology Ethics Committee, and all those taking part provided written consent for their participation.

#### Apparatus and Materials

Information associated with four different knots, including step-by-step pictorial knot-tying instructions, was obtained from www.animatedknots.com. The two knots specifically selected for their simplicity were the Carrick Bend (a boating knot), and the Improved Clinch (a fishing knot), and the two selected for their difficulty were the Purcell Prusik Loop (a rescue knot) and the Trucker’s Hitch (a boating knot). The knots were selected partly according to the number and diversity of the steps involved in tying the knot, and partly according to subjective judgment of perceived difficulty by the experimenter. The step-by-step instructions available on the website consisted of a sequence of pictures of the knot in question in various stages of completion, and the number of pictures was, as might be expected, also approximately in line with the simple/complex categorisation (Carrick Bend 8; Improved Clinch 10; Purcell Prusik Loop 13; Trucker’s Hitch 11). For three of the knots (Carrick Bend, Improved Clinch and Purcell Prusik Loop) the final step(s) simply involved tightening the knot, and therefore produced a version of the knot that would have been scored as technically correct. Technically correct (but looser) versions were depicted in Step 6 (Carrick Bend), Step 9 (Improved Clinch) and Step 11 (Purcell Prusik Loop).

Participants were provided with string(s) cut to lengths appropriate to the knot they were being asked to tie, in colours that were a close match to those represented in the relevant target images. They were also provided with metal rings if these were depicted in the images of the relevant knot.

**Fig. 1:**
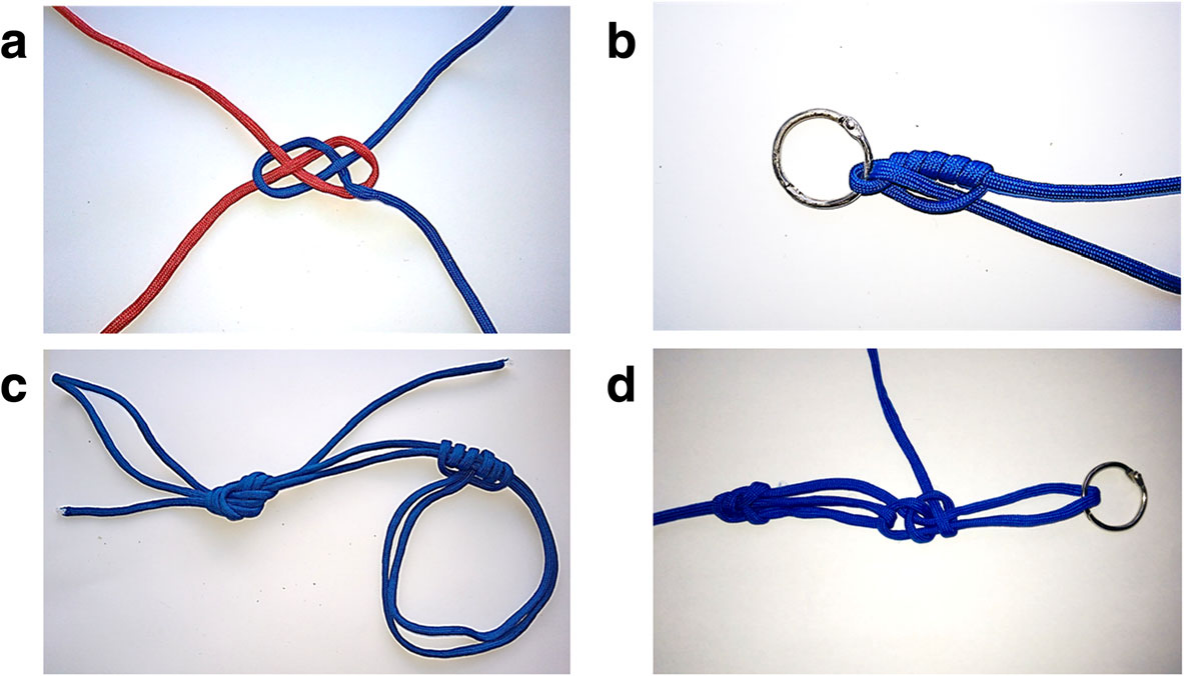
Examples of the knots used in this study, completed by the experimenter (CC) using the materials provided to participants. Upper panels display the less challenging knot types (**a** Carrick Bend, and **b** Improved Clinch), and lower panels the more complex examples (**c** Purcell Prusik Loop, and **d** Trucker’s Hitch)

Example images of each of the four completed knots, created using the materials from the current study, can be seen in Fig. 1. The original images used as stimuli can be viewed at www.animatedknots.com. For the purpose of the current experiment the step-by-step pictorial instructions displaying the intermediate states were bound together in the form of a booklet for each of the different knots. The images depicted only string and metal rings. No human body parts (e.g. hands holding the string) were included in these images. The image of the Carrick Bend knot depicted in Fig. 1 represents the image shown in Step 6 of the instructions from animatedknots.com (loose version, see above regarding the number of steps involved in tying each knot), as this was the standalone image used to illustrate the knot on the website. This was therefore the single image provided to participants in the End State Only condition (see Procedure). The completed knot provided to participants, in contrast, was a fully tightened version of the Carrick Bend. For the other three knots the image used to illustrate the knot was the same as the (fully tightened) final image in the step-by-step image sequence.

It should be noted that the knots differed in many respects other than just their relative difficulty. Two incorporated metal rings (as shown in Fig. 1), and one made use of two different string colours. The knots are also used for a range of different purposes. Their functions are listed as follows on the animatedknots.com website: boating (Carrick Bend and Trucker’s Hitch), fishing (Improved Clinch), and rescue (Purcell Prusik Loop). It is possible that these factors may have influenced the ease of execution and/or learnability of the knots, although this was not systematically investigated.

A camera was used to record each participant’s attempt at their assigned knot during the Test Trial.

#### Procedure

Each participant was recruited from one of ten practical class sessions (each comprising between 13 and 17 students), and all participants in a given class took part in the experiment at the same time. At the start, images of the four knots were displayed using an overhead projector, and all participants confirmed that they had no prior knowledge of how to construct any of the knots. Participants were not questioned regarding their general background experience of knot-tying, beyond these specific examples.

##### Practice Trial: Assigning Teacher Role

This initial knot-tying phase was used to determine which participants would be assigned the role of teacher for the experimental comparison between different learning conditions (the *Test Trial*). The experimenter assigned each participant a knot, equally distributed throughout the class to ensure an approximately equal assignment of each of the different knot types, and to ensure that no individual was sitting close to another participant who had been assigned the same knot. This was achieved by cycling through all four different knot types in the same repeating order, as materials were handed out to participants. This ensured that participants could not be sitting next to someone else who was assigned the same knot type as themselves.

Each participant was provided with the learning materials appropriate to their target knot. This consisted of an example completed knot which had been tied by the experimenter (and which they were not permitted to untie), the booklet of intermediate state images intended to guide them through the process of tying the knot, and the image of the completed knot. In order to tie their own knots, participants were also provided with string(s) of appropriate length, and metal rings if required.

Since the aim of this phase was to identify suitable teachers, rather than evaluate performance, participants were permitted to request informal help from the experimenter if they felt this would help them learn to tie their assigned knot. In each practical class, the goal of the Practice Trial was to identify at least one suitable teacher per knot type (to give a total of 40 teachers across all classes; ten per knot). When all four of the knots had each been mastered by at least one individual (and those participants had agreed to take on the role of teacher for the next phase), the Practice Trial was concluded and all knot-tying materials and learning aids were collected from participants. To be assigned the role of teacher, participants were required to have successfully completed their knot at least once. In the subsequent phase of the experiment (Test Trial, see below) those assigned the role of teacher were only ever expected to teach the particular knot for which they had demonstrated mastery during the Practice Trial.

##### Test Trial: Comparing Learning Conditions

Participants not assigned the role of teacher were provided with a new knot to learn, and were assigned to one of the three different learning conditions: End State Only; Intermediate States; and Teaching. Members of the class assigned the role of teacher swapped places with other individuals to ensure they were seated next to their assigned learner. Learners were assigned to knot types, and learning conditions, such that (as in the Practice Trial) they were not sitting next to another participant attempting to learn the same knot. Thus assignment to knot type and learning conditions was not random. However, it was done in such a way that there should not have been systematic differences between either knot type conditions or learning conditions (e.g. in terms of seating position in the class). Learners that had been assigned one of the simple knots during the Practice Trial were assigned one of the complex ones for the Test Trial, and vice versa. This was for the purely pragmatic motive that as many participants as possible should have the opportunity to attempt knots of both levels of difficulty, given the practical class context.

All participants were provided with the materials needed to tie their knot. In the End State Only condition, participants were also provided with an example completed knot, along with the image of the knot. In the Intermediate States condition, participants were provided with the booklet of intermediate steps for their knot, as well as the completed knot and image of the knot. In the Teaching condition, in addition to the same materials provided to those in the Intermediate States condition, the teacher-learner pairs were provided with an extra set of knot-tying materials for the teacher. Therefore, teachers were potentially able to use their own set of materials to demonstrate the process for the learner, as well as giving verbal instruction and feedback. Teachers were however informed that they should not under any circumstances interfere with their assigned learner’s set of materials, which were to be used by the learner alone. This was to ensure that teachers did not complete any part of the knot that was to be recorded as the learner’s attempt. In other respects, the teachers were permitted to teach the knot in any way they saw fit. We regarded this as very important given our logic that the potential additive benefits of teaching (over and above process information) were most likely to arise as a consequence of the flexibility of the teachers’ behaviour allowing for responsiveness to the learners’ needs. So it was critical to ensure that they were permitted this flexibility.

During the Test Trial, participants were given up to 30 min to tie their knot. By the end of this period, most participants had already completed their attempt, and a photograph had been taken by the experimenter. However, those who were still engaged in the task were asked to leave their attempt as close to completion as they had been able to achieve, and these partially completed attempts were recorded in the same way. Photographs were taken with knots oriented such that their structure was clear and they could be readily compared with the target images, as far as was possible.

Three of the 115 knots tied by participants in the Test Trial were not successfully recorded on camera. All were from the End State Only learning condition. Two were not photographed due to experimenter error (one Carrick Bend and one Improved Clinch) but the attempts were recorded on paper as having been entirely successful. The remaining knot (a Purcell Prusik Loop) was not photographed due to the participant electing to submit no attempt, as a result of having given up on the task. This attempt was recorded on paper as entirely unsuccessful.

#### Data Coding

Task success was measured by comparing the photographs of the produced knots to their target images. Coding was carried out by the same cohort of students in a subsequent series of practical classes. Photographs were identified by unique anonymous participant codes. Raters therefore had no way of knowing the identity of the knot’s creator, nor which of the learning conditions that individual had been assigned. The experimenter, who was experienced in tying the knots as a result of running the classes, also rated the photographs in the same way (i.e. blind to participant identity and learning condition).

Raters were asked to give the knots a “success score” between 1 and 5, such that: “5 indicates that the knot is complete with no mistakes. 4 indicates that the knot is more than half complete and correct. 3 indicates that the knot is about half complete and correct. 2 indicates that the knot is less than half complete and correct. 1 indicates that no part of the knot has been completed.”

Each knot photograph was rated by between five and 11 different coders. Each student coder rated only a small subset of the photographs, but the experimenter (CC) rated the full set. Student ratings showed a high concordance: from a total of 942 individual (student) ratings, 699 (74%) were the same as the median rating for that knot. These median scores were therefore taken as the value representing the student coders’ rating of each knot. There was very high correlation between the median student rating and the experimenter’s rating for each photograph (*r* = 0.926, *N* = 112, *p* < .001). The overall median rating (i.e. including the experimenter as one of the coders for every knot) was used for further analysis. The informal records of knot success taken during data collection were used for the three knots for which there was no photograph (with the successfully completed knots converted to scores of 5, and the completely unsuccessful attempt converted to a score of 1).

### Results

**Fig. 2:**
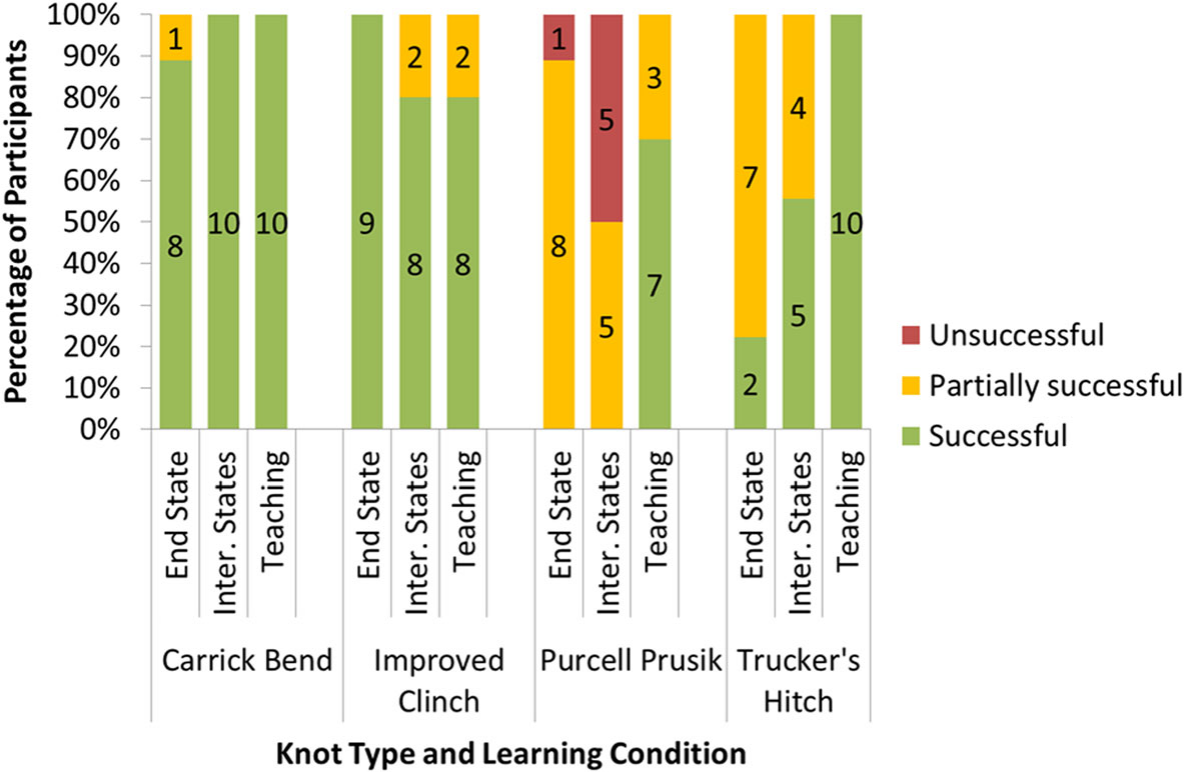
Percentage of participants who were successful (scores of 5), unsuccessful (scores of 1), or partially successful (all other scores), depending on knot type and learning condition. Numbers on bars indicate number of participants in each category

Mean success scores for all four knots, for the three different learning conditions, are displayed in Table 1. Figure 2 shows how many participants were successful (scores of 5), unsuccessful (scores of 1), or partially successful (all other scores), depending on knot type and learning condition.

**Table 1:** Mean success scores (using a five-point rating scale) for the four knot types, under the three learning conditions. Standard deviations are given in brackets

Learning Condition	Knot Type							
	Carrick Bend		Improved Clinch		Purcell Prusik Loop		Trucker’s Hitch	
End State Only	4.78	(0.6)	5.00	(0)	2.67	(1.12)	3.33	(1.22)
Intermediate States	5.00	(0)	4.85	(0.34)	2.00	(1.05)	4.33	(0.87)
Teaching	5.00	(0)	4.80	(0.42)	4.60	(0.70)	5.00	(0)

Knot success scores were analysed using linear regression (R Core Team 2013; Bates et al. 2013), with knot complexity (simple, i.e. Carrick Bend or Improved Clinch; or complex, i.e. Purcell Prusik Look or Trucker’s Hitch), learning condition, and their interaction as fixed effects, and practice knot, practice success, test knot, and class group, included as random intercepts. Complex knots in the End State Only condition were taken as the baseline, and *p*-values were estimated from the resultant t-statistics with degrees of freedom being the number of observations minus the number of fixed parameters in the model (Baayen et al. 2008). The model was significantly better than its null equivalent (χ2(5) = 63.322, *p* < .001). There were significant effects of knot complexity (β = 1.899, SE = 0.583, t(110) = 3.257, *p* = .001), the Teaching condition (β = 1.799, SE = 0.240, t(110) = 7.486, *p* < .001), and the interaction between knot complexity and the Teaching condition (β = −1.790, SE = 0.340, t(110) = −5.266, *p* < .001). There was no effect of the Intermediate States condition or the interaction between knot complexity and the Intermediate States condition (β ≤ 0.031, SE ≥ 0.250, t(110) ≤ 0.124, *p* ≥ .902). A Tukey multiple comparison of means indicated a significant difference between the Teaching and End State Only conditions (β = 1.799, SE = 0.240, z = 7.486, *p* < .001), and the Teaching and Intermediate States conditions (β = 1.768, SE = 0.244, z = 7.242, *p* < .001), but no difference between the Intermediate States and End State Only condition (β = 0.031, SE = 0.250, z = 0.124, *p* = .992).

### Conclusions

The results permit a number of conclusions. Firstly, our classification of the knots as “simple” and “complex” was supported by the analysis showing that, overall, success scores were significantly lower for the knots classified as complex, compared with those classified as simple. Also, in line with predictions, the Teaching condition was the most effective means of transmission of knot-tying skills for those knots exhibiting differences in success scores across conditions. Specifically, it was found that pairing a naïve learner with an experienced partner was more effective than learning from end state information alone, and that this effect was more apparent for knots that were classified as more challenging and complex. The Teaching condition was also more effective than the Intermediate States condition, in which participants were provided with a series of static images illustrating the process of completion in a step-by-step fashion. This suggests that having access to an experienced teacher provides benefits over and above exposure to stages of the process (as one might obtain as a consequence of contact with experienced individuals in the absence of teaching).

It should be noted that due to the group context in which the data were collected, we cannot completely rule out the possibility that some participants may have been able to catch glimpses of others some distance away (although never in the adjacent seat, see Methods) who were completing the same knot they had been assigned. However, if so then this would have been consistent across conditions, and therefore should not confound comparisons between conditions. Also, in terms of the potentially beneficial effects of (experimentally uncontrolled) opportunities to observe someone else completing the knot, over and above having access to the learning materials provided, this would have been greatest for the End State condition, and most negligible for the Teaching condition. Consequently any such issues would be unlikely to cast doubt on our conclusions, as they would tend to weaken differences between conditions, leading us to underestimate, if anything, the size of any differences.

The enhanced performance observed in the Teaching condition, relative to the other learning conditions, may be due to teachers’ ability to be responsive to the needs of learners, which would allow them to provide feedback on the learner’s attempts, and/or target their own instructive behaviour towards addressing any apparent shortcomings in the learner’s understanding or performance. Neither source of information would have been available from the step-by-step instructions. It should be noted however that this remains a speculative conclusion on the basis of the current dataset, since it was not possible to record the nature of the interactions and communication that occurred between teachers and learners. We believe that future research recording and analysing spontaneous teaching behaviour would be extremely valuable, and would shed light on the information available to learners within such interactions, as compared with non-interactive exposure to process information.

In contrast with the complex knots, the simple knots were reproduced with high fidelity under all conditions. Although it was expected that teaching would increase success rates more for the complex knots compared with the simpler alternatives, this must remain a relatively cautious conclusion based on the current data. Success scores were so high for the simple knots that it was impossible to determine whether teaching also offered some benefits to transmission of these more transparent skills, as this may simply not have been detected here due to participants’ ceiling level performance. Nonetheless it can certainly be concluded that there was clear evidence that transmission of the more complex knots was facilitated in the teaching condition, whereas this was not apparent for the simpler types.

In relation to this point, it should be emphasised that investigating this relative degree of benefit from teaching for the simple and complex knots was really the key motivation for the current study. The learning conditions, as operationalised in the current study, incorporated sources of information in an additive fashion. So individuals in the Teaching condition had access to the same materials as those in the Intermediate States condition, in addition to being paired with an experienced partner. Similarly, participants in the Intermediate States condition had access to the same materials as were available to those in the End State Only condition, but with the addition of the step-by-step instructions. Consequently, it should not come as a surprise that more information led to more effective learning. However, the fact that it did so apparently exclusively for the complex knots provides insights into the conditions under which teaching offers particular benefits. Another fruitful avenue for future research might attempt to isolate the effects of information available from intentional teaching from the effects of information available from observation. This could be achieved by, for example, permitting only remote communication between partners. This would make it possible to identify factors influencing whether behaviours were more effectively transmitted via observation in the absence of teaching, or alternatively, via teaching in the absence of observation.

## General Discussion

Returning to the debate reviewed in the introduction, the study reported here (in combination with the other experimental work reviewed in Section 1.4) does shed some light on the topic, although the limitations of such approaches must not be overlooked (and we return to consider these below). In showing that cumulative cultural evolution can arise from learning from end products alone, Caldwell and Millen’s (2009) results certainly cast doubt on the notion of teaching as an essential precursor, and this is reinforced by Zwirner and Thornton’s (2015) replication of this finding using a different task. This would imply that some degree of cumulative culture could have existed in hominin populations without these individuals exhibiting propensities for teaching on a par with modern humans. Therefore, of the two alternative unidirectional proposals, it seems to us more plausible that the existence of a rudimentary form of cumulative culture might have either generated the selective advantage required for specialized capacities for teaching to evolve (e.g. Csibra and Gergely 2011), or enabled the cumulative cultural evolution of teaching behaviours themselves (e.g. Heyes 2016b). The evidence from the study reported above lends some additional weight to this idea, since it is clear from these results that teaching can facilitate transmission of skills. Indeed, the results also suggest that such benefits may be most apparent for relatively advanced skills. These are likely to represent outcomes of more extended sequences of accumulation of modifications, which rendered them relatively opaque in terms of the actions required for successful completion.

However, it should be noted that the evidence is not at odds with the two remaining proposals raised in the introduction (i.e. bidirectional coevolutionary causation, and alternative causal influence enabling both capacities). Furthermore, it must also be emphasized that the existing studies can contribute little to the question of the selective pressure (if indeed there was some key event or set of conditions at all) which triggered the development of the precursor trait or traits. Therefore, regardless of which of these interpretations we might favour, questions remain which the experimental evidence cannot speak to: what could have caused early hominins to develop cultural contents more complex than their predecessors and contemporaries, if this was not an outcome of the presence of particular transmission capabilities?; if teaching and cumulative culture are so inextricably linked by mutual influence as to render it impossible to identify one as more fundamental than the other, then what could have prompted the emergence of such a complement?; and if a third factor (such as social metacognitive capacities) enabled both, then is it possible to identify some selective pressure for *this* trait? It is well beyond the scope of the current manuscript to speculate on such matters, although we note that these questions have been addressed elsewhere (e.g. see Richerson and Boyd 2005, for a proposal linking the evolution of human cultural capacities to Pleistocene climate fluctuations).

In addition, regardless of whether teaching is concluded to be a key precursor of cumulative culture, it should be emphasised that it would be – at most – only one of several such precursors. To provide just two likely examples, the manifestation of cumulative culture has been proposed to be dependent on demographic factors, such as population size and structure (e.g. Derex and Boyd 2016), and technological advances will clearly be dependent on learners having some understanding of physical causality (e.g. Osiurak et al. 2016).

It is also important to note that the study presented in the current manuscript, in contrast to the studies of Caldwell and Millen (2008, 2009), is not a study of cumulative culture per se, since no attempt was made to track improvement in learner performance over multiple generations. The experiment reported here was therefore only a study of the effectiveness of transmission (and hence prevention against loss, rather than improvement). Within the literature on human cultural evolution, the emphasis has typically been placed on the importance of high fidelity transmission, and even blind copying (e.g. Lewis and Laland 2012; Tennie et al. 2009). However, faithful transmission alone is unlikely to result in cumulative culture as we have conceptualized it here. The nature of the innovative element involved in the process of cultural evolution may have a key influence on the outcome of that process (Caldwell et al. 2016). It is quite likely that innovative processes in humans have been relatively overlooked as a potentially significant factor in the distinctiveness of human culture.

In conclusion, teaching behaviour (as manifested in humans) appears strikingly well-designed for the transmission of cumulative culture. Flexibility enables the transmission of novel traits, and a high level of responsiveness to the perceived needs of the learner particularly facilitates the transmission of hard-to-learn skills. Furthermore, it appears that even very young children selectively focus on hard-to-learn skills when teaching others (Ronfard et al. 2016). The evolutionary history of these two features may therefore be linked. Experimental studies of behavioural transmission and accumulation can provide a key source of evidence in understanding this relationship through the manipulation of the availability of sources of information. In this way it is possible to determine limits on transmission and cumulative improvement under different conditions. Future research using such approaches may be able to identify relevant generalizable factors which distinguish the types of skill which can be preserved and/or enhanced under different transmission conditions.
